# Developing electrical properties of postnatal mouse lumbar motoneurons

**DOI:** 10.3389/fncel.2015.00349

**Published:** 2015-09-02

**Authors:** Jacques Durand, Anton Filipchuk, Arnaud Pambo-Pambo, Julien Amendola, Iryna Borisovna Kulagina, Jean-Patrick Guéritaud

**Affiliations:** ^1^Institut de Neurosciences de la Timone, Aix Marseille Université – CNRS, UMR 7289Marseille, France; ^2^International Center for Molecular PhysiologyKiev, Ukraine

**Keywords:** spinal, discharge firing pattern, dendritic arborization, calcium

## Abstract

We studied the rapid changes in electrical properties of lumbar motoneurons between postnatal days 3 and 9 just before mice weight-bear and walk. The input conductance and rheobase significantly increased up to P8. A negative correlation exists between the input resistance (Rin) and rheobase. Both parameters are significantly correlated with the total dendritic surface area of motoneurons, the largest motoneurons having the lowest Rin and the highest rheobase. We classified the motoneurons into three groups according to their discharge firing patterns during current pulse injection (transient, delayed onset, sustained). The delayed onset firing type has the highest rheobase and the fastest action potential (AP) whereas the transient firing group has the lowest rheobase and the less mature AP. We found 32 and 10% of motoneurons with a transient firing at P3–P5 and P8, respectively. About 20% of motoneurons with delayed onset firing were detected at P8. At P9, all motoneurons exhibit a sustained firing. We defined five groups of motoneurons according to their discharge firing patterns in response to ascending and descending current ramps. In addition to the four classical types, we defined a fifth type called transient for the quasi-absence of discharge during the descending phase of the ramp. This transient type represents about 40% between P3–P5 and tends to disappear with age. Types 1 and 2 (linear and clockwise hysteresis) are the most preponderant at P6–P7. Types 3 and 4 (prolonged sustained and counter clockwise hysteresis) emerge at P8–P9. The emergence of types 3 and 4 probably depends on the maturation of L type calcium channels in the dendrites of motoneurons. No correlation was found between groups defined by step or triangular ramp of currents with the exception of transient firing patterns. Our data support the idea that a switch in the electrical properties of lumbar motoneurons might exist in the second postnatal week of life in mice.

## Introduction

Electrical properties of developing spinal motoneurons have been studied in several species and at different embryonic and postnatal stages (Ziskind-Conhaim, 1988; Navarrette and Vrbová, 1993; Perrier and Hounsgaard, 2000; Vinay et al., 2000a,b, 2002; Carrascal et al., 2005; Kanning et al., 2010). Only a few studies deal with developing mouse spinal motoneurons (Mynlieff and Beam, 1992a,b; Nakanishi and Whelan, 2010; Quinlan et al., 2011). A new interest to study the development of electrical properties in mouse motoneurons came with the discovery that spinal motoneuron pathology starts during the postnatal period in superoxide dismutase 1 (SOD1) transgenic mice, a standard model of amyotrophic lateral sclerosis (ALS; Amendola et al., 2004, 2007; Durand et al., 2006; Bories et al., 2007; Amendola and Durand, 2008; Pambo-Pambo et al., 2009; Quinlan et al., 2011; Filipchuk and Durand, 2012; Saxena et al., 2013).

The functional differentiation into fast and slow-twitch muscle fibers takes place late in embryonic and early in postnatal life and depends on the properties of the motoneuron (Buchthal and Schmalbruch, 1980; Navarrette and Vrbová, 1993; Kanning et al., 2010). Poly-innervation and gap junctions are present at that time period when motoneurons are still competing at the periphery (Navarrette and Vrbová, 1993; Kopp et al., 2000; Vinay et al., 2000b; Pun et al., 2002) precluding a functional identification of motor units *in situ*. For example, 64% of neuromuscular junctions mouse soleus muscle are multiply innervated by P7 whereas about 43% of the junctions are still innervated by two or more axons at P9 (Kopp et al., 2000). At that time, the firing patterns of soleus motor units are “quite phasic” (Navarrette and Vrbová, 1993; Kopp et al., 2000; Personius and Balice-Gordon, 2002).

During the early postnatal period, three patterns of discharge firing (single, transient and sustained) following current pulse stimulation have been well documented in rat spinal motoneurons (Vinay et al., 2000a,b, 2002; Mentis et al., 2007). In addition, a delayed onset firing type was recently described in mouse spinal motoneurons (Pambo-Pambo et al., 2009; Leroy et al., 2014). This delayed onset firing is due to transient outward potassium currents (Takahashi, 1990; Russier et al., 2003; Pambo-Pambo et al., 2009). It was observed in postnatal abducens motoneurons during a precise postnatal period between P4 and P9 (Russier et al., 2003). In this study we investigated whether the different patterns are present in mouse lumbar motoneurons at the same age and we focused on the postnatal period P3–P9 when pathological signs have been observed in the spinal cord of SOD1 mice (Bories et al., 2007; Amendola and Durand, 2008; Filipchuk and Durand, 2012; Saxena et al., 2013). We also analysed the development of the delayed onset firing type in spinal motoneurons to determine whether it disappears in the second postnatal week as in the case of abducens motoneurons.

Correlation between rheobase and input resistance (Rin) of motoneurons has been found in the neonate rat (Seebach and Mendell, 1996). Indeed, we investigated the correlations between the size of motoneurons and both parameters (rheobase and Rin) using our database on mouse lumbar motoneurons that have been intracellularly recorded and stained with Neurobiotin at both ages P3–P4 and P8–P9. We also compared several parameters at two different postnatal ranges (P3–P5 and P8–P9) to detect rapid changes during this period and to supplement previous studies on mouse lumbar motoneurons (Nakanishi and Whelan, 2010; Quinlan et al., 2011). Finally, we investigated the development of repetitive firing and the electrical properties of mouse lumbar motoneurons in the different groups sorted by their firing patterns. We found three types of discharge firing patterns using current step stimulation. Surprisingly in a recent study, only two patterns of discharge firing were described in mouse lumbar motoneurons in postnatal mouse (Leroy et al., 2014). Four firing patterns were previously found with ascending and descending current ramp stimulation (Amendola et al., 2007; Pambo-Pambo et al., 2009). In this study we defined a fifth type (transient firing) and we determined the ratio of motoneurons in the different types.

Part of this work has been published in abstract form (Durand et al., 2013).

## Materials and Methods

Experiments were carried out on C57BL/6J mice aged from postnatal day 3 (P3) to 9 (P9), P0 being the first postnatal day. All surgical and experimental procedures are conformed to the European Communities council directive (86/609/EEC) and approved by our ethics committee (Comité National de Réflexion Ethique sur l’Expérimentation Animale n° 71). Most of the experimental procedures were described previously (Bories et al., 2007; Amendola and Durand, 2008).

### Electrophysiological Experiments

P3–9 pups were anesthetized by hypothermia, decapitated, eviscerated and pinned down onto a Petri dish and immersed in cold (4°C) artificial cerebrospinal fluid (ACSF). Then, a laminectomy was performed and the spinal cord and brainstem were removed, taking care to preserve sufficient length of L5 ventral root, placed in a recording chamber and superfused with ACSF containing (in mM): NaCl, 130; KCl, 4; MgCl_2_, 1.2; CaCl_2_, 2; NaH_2_PO_4_, 1; NaHCO_3_, 25; D-glucose, 30; bubbled with a 95% O_2_*/*5% CO_2_ mixture, adjusted to pH 7.4 at 24–25°C. Monopolar stainless steel electrodes were placed in contact with the L5 ventral root and insulated with petroleum jelly for recordings and simulations.

To allow for microelectrode penetration in the spinal cord, the pia was carefully removed medially to L5 ventral root entry, using very fine forceps under binocular control. Fine tip micropipettes for intracellular recordings were made from 1.2 mm filamented glass tubes (Clark Instruments) using a pipette puller (model P-97; Sutter Instruments). Electrodes were filled with 2 M potassium acetate, and their resistances ranged between 60 and 110 MΩ. The microelectrode was positioned to penetrate the L5 spinal segment with an angle of 30–45° above the horizontal and advanced in the tissue using a Narishige^TM^ three-dimensional hydraulic microdrive. Motoneurons were impaled at a depth of 150–450 μm from the spinal cord surface corresponding to the fifth lumbar segment. Motoneurons were identified by their antidromic action potential (anti AP) evoked following electrical stimulation of the ventral root L5. Intracellular recordings were made either in bridge mode with an output bandwidth of 3.0 kHz or in Discontinuous Current Clamp (DCC) mode, using an Axoclamp 2B amplifier (Axon instruments). Electrode resistance and capacitance were compensated before intracellular recordings. Signals were digitized at 10 kHz by an A/D converter (Digidata 1322 Axon Instruments) and saved on a computer using Clampex 9.2 (Axon instruments).

### Data Analysis

Rin was measured by computing the voltage deflections derived from series of hyperpolarizing and depolarizing constant current pulses (350 ms; −0.4 nA to +0.4 nA) injected into the motoneurons. Measurements were made from the averaged voltage over 50 ms taken from the steady state membrane potential at the end of the pulses. Retained values were the averages of three sets of measurements.

Spike potentials were analyzed using the Event Detection/Threshold Search module of Clampfit 9.2. Firing behavior was studied as described previously (Bories et al., 2007). Briefly, we used intracellular injection of series of depolarizing constant current pulses of increasing amplitude, 800 ms to 1 s in duration. Frequency/current relationship was determined as the slope of the regression line fitted to the F-I curve in the steady state (last 500 ms). A second protocol using triangular current injection was performed to study the firing pattern, instantaneous frequency and F-I relationship following ascending and descending ramps of current (Amendola et al., 2007). The triangular current stimulation consisted in an ascending followed by a descending current command. Both ramps were symmetric and series of ramps were performed every 30 s each with a speed between 0.25 nA/s to 0.95 nA/s. The different types of F-I patterns depend upon comparing the F-I curve when current is increasing to that obtained when current is decreasing (Hounsgaard et al., 1988).

Statistical significance was assessed with the non-parametric Permutation with General Score Exact Test for independent or paired samples or the Fisher exact test (StatXact7. Cytel software). The correlation between two sets of data was evaluated by using Pearson’s correlation test or Spearman’s correlation test (Graphpad Prism 6 or StatXact 7). Two groups of data were considered statistically different (*) if *p* < 0.05, the difference being highly significant (**) if *p* < 0.01 or (***) if *p* < 0.005. Results were expressed as means ± SEM or medians with interquartile range when indicated. Graphical representations were obtained using Origin 7.5 (Origin Lab Corporation), Graph pad Prism 6.0 and Corel Draw 12 (Vector Capital, San Francisco, CA, USA).

### Labeling of Motoneurons

All the procedures for morphological studies were described previously (Amendola and Durand, 2008; Filipchuk and Durand, 2012). Labeling of recorded motoneurons was done by intracellular injection of 2% Neurobiotin following recording sessions. The motoneurons were stained using depolarizing current pulses (duration 150 ms, 1–4 nA) applied at 3.3 Hz for 10–20 min. After Neurobiotin injection, the spinal cord was maintained in the recording chamber for 1–2 h to allow for diffusion of marker into distal dendrites. The spinal cord was then immersed in 4% Paraformaldehyde fixative at 4°C overnight, rinsed in PBS (pH 7.4), blocked and cut transversally at 75 μm on a vibratome (Microm HM 650V). Neurobiotin was revealed using the standard avidin-HRP-diaminobenzidin staining procedure. Serial sections were mounted on gelatin covered glass slides, air dried overnight and coversliped.

### Quantitative Morphometric Analysis

The labeled motoneurons were reconstructed from serial sections (75 μm thick) on Nikon microscope equipped with a computer interfaced motorized stage and *z*-axis optical encoder using Neurolucida^TM^ software. The Nikon microscope was equipped with ×20 dry objective and a numerical zoom ×3 (final magnification ×60).

A single motoneuron was described by up to 18.000 data points, which were stored in a database together with fiducial marks (boundaries of transverse spinal cord sections and central canal) in ASCII format files. Reconstructed cells were visualized and three-dimensionally analyzed using Neurolucida^TM^. Our own database of intracellularly recorded and stained motoneurons with Neurobiotin comprises more than 50 postnatal mouse lumbar motoneurons at different ages between P3 and P10. Among them, 32 motoneurons were fully reconstructed in 3D with Neurolucida^TM^. In the present work we used 14 motoneurons at both ages (P3–P4, *n* = 3 and P8–P9, *n* = 11).

## Results

**Figure 1:**
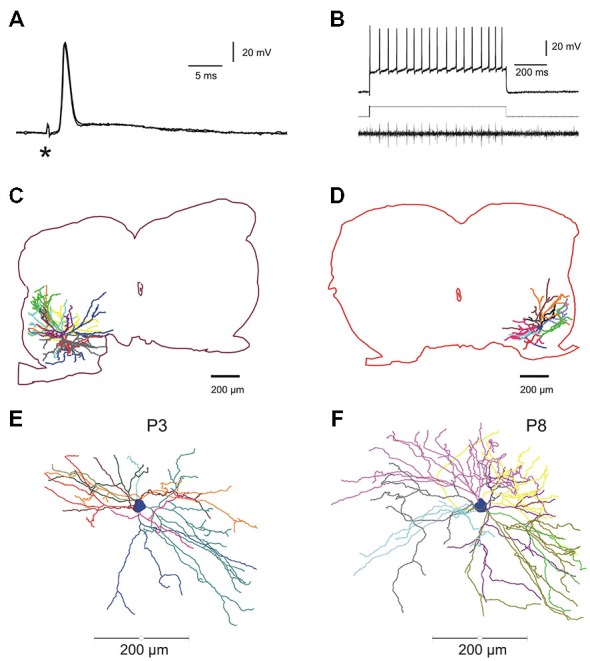
**Electrophysiological identification and intracellular staining of lumbar motoneurons in the developing mouse spinal cord. (A)** Electrical stimulation of the fifth lumbar (L5) ventral root evoked an anti AP. Asterisk indicates the stimulus artifact. **(B)** Direct intracellular stimulation of L5 motoneuron giving rise to a train of action potentials (APs; sustained discharge) recorded intracellularly (upper trace) and the propagated spikes in the ventral root (lower trace); rectangular injected current: 1.2 nA (middle trace). **(C,D)**, two fully reconstructed lumbar motoneurons recorded from P8–P9 mice. Depending on the location of the soma in the ventro-lateral part of the spinal cord, dendritic arborisations extended either in all rostro-caudal directions and medially near the central canal **(C)** or confined in a restricted area into the latero-ventral part of the spinal cord **(D)**. **(E,F)** Digitized full reconstructions of two motoneurons at postnatal days 3 and 8 (P3 and P8) in the transverse plane. During this period, the total dendritic length increases by 22%, only. Each dendrite is represented by a specific color.

**Figure 2:**
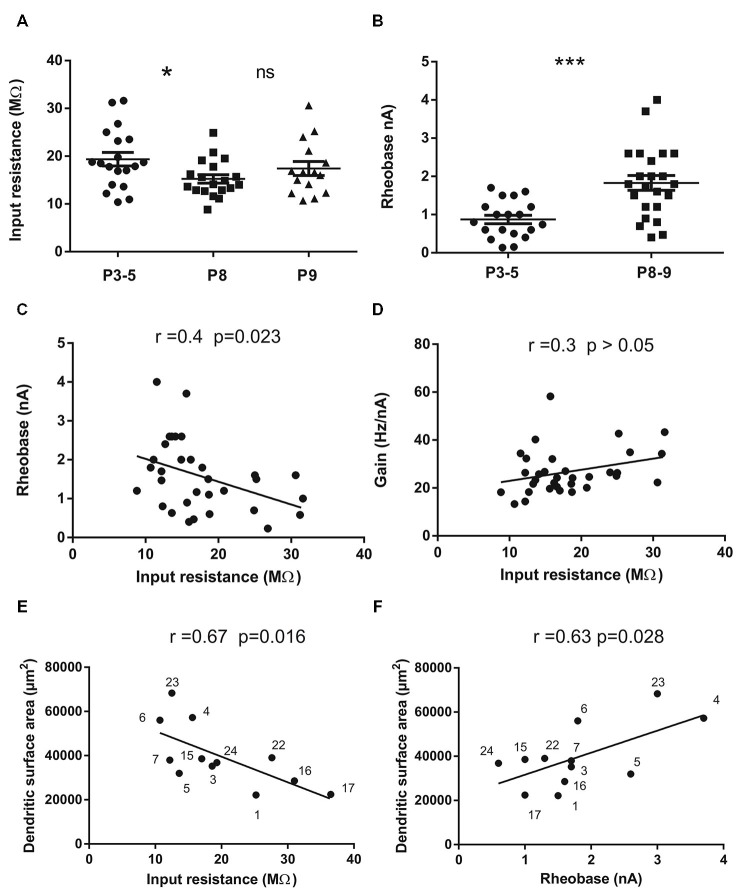
**Input resistance (Rin) and rheobase of mouse lumbar motoneurons (*n* = 53) at different postnatal ages (P3 to P9) and correlations with total dendritic surface area in 12 motoneurons stained with neurobiotin. (A)** Mean Rin significantly decreases between the groups P3–5 and P8 (*p* = 0.028, *n* = 19 in each group) and then stabilizes at P9 (*n* = 15). **(B)** the mean rheobase (minimum current injected into a neuron to elicit an AP in 50% of cases) increases in the same period of time (*p* = 0.0033, *n* = 19 for P3–5 and *n* = 31 for P8–9). **(C)** significant negative correlation exists between rheobase and Rin in the whole population (*n* = 32); **(D)** no significant correlation was found between the Rin and the gain of the motoneurons (*n* = 32). The gain is the slope of the F-I curves measured in the steady state of the discharge firing elicited during rectangular pulses of currents. **(E,F)** significant correlations between Rin **(E)** rheobase **(F)** and the total dendritic surface area measured using 3D reconstructed motoneurons with neurolucida. Each motoneuron is numbered so that it can be identified from previous publications Amendola and Durand (2008) for Mn n° 1–7 and Filipchuk and Durand (2012) for Mn n° 15, 16, 17. The morphologies of motoneurons n° 22, 23 and 24 were not previously published. The largest motoneurons tend to have the lowest Rin and the highest rheobase. Horizontal bars indicated mean ± sem in the scatter plots of A and B. For statistical significance nonparametric permutation or mann-whitney exact tests and Pearson’s correlation test were used. *p < 0.05; **p < 0.01; ***p < 0.005. Non-significant (ns) *p* > 0.05.

The data base for the electrophysiological study comprises 103 motoneurons from mice aged between P3 and P9. Only neurons displaying a stable membrane potential more negative than −50 mV with overshooting APs during the whole test procedure were kept for analysis. All the motoneurons were identified by recording either the anti AP evoked by the ventral root stimulation (Figure 1A, two superposed traces) or the orthodromic AP in the ventral root (Figure 1B, VR lower trace) evoked by direct intracellular stimulation (Figure 1B, upper trace). Among them, 12 motoneurons intracellularly recorded and stained were taken from our library of 3D reconstructed lumbar mouse motoneurons to link the electrical parameters (rheobase, Rin) and the morphology of individual lumbar motoneurons (see below and graphs; Figures 2E,F).

### Morphological Changes Between P3 and P9

Most of the results on morphological data were published elsewhere for 9 out of 12 motoneurons (Amendola et al., 2007; Amendola and Durand, 2008; Elbasiouny et al., 2010a; Filipchuk and Durand, 2012). The dendritic arborizations elongated between P3 and P9 without increasing their number of branches (Filipchuk and Durand, 2012). The soma size significantly increases with age (P3–4: 1864 ± 120 μm^2^, *n* = 3; P8–9: 3212 ± 314 μm^2^, *n* = 9; *p* = 0.04) as well as the mean diameter of primary dendrites (P3–4: 3.56 ± 0.32 μm^2^, *n* = 3; P8–9: 5.07 ± 0.31 μm^2^, *n* = 9; *p* = 0.03). The total dendritic length increases from P3 to P9 by 22% (P3–4: 13622 ± 2468 μm; P8–9: 16658 ± 1625 μm; non-significant difference, ns) and the total dendritic surface area by 20% (Filipchuk and Durand, 2012). Depending on the soma location in the ventro-lateral part of the spinal cord, distal dendrites reached different distal zones. When the soma was located centrally and dorsally in the ventro-lateral region, the dendrites projecting medially reach a region close to the central canal known to contain premotor interneurons (Figure 1C). On the contrary the dendritic extension of motoneurons was restricted when the soma was situated in close proximity to the ventral horn boundaries (Figure 1D). The full 3D reconstructions of the whole dendritic arborizations include the rostro-caudal extensions (between 450 and 750 μm) and illustrate the relative complexity of the single trees at both ages P3 and P8 (see also Figure 3 in Amendola and Durand, 2008).

### Electrical Properties of Mouse Lumbar Motoneurons: Correlations with Size

In this series of experiments, a number of parameters of electrical properties was analyzed in two populations of lumbar motoneurons of different ages (P3–P5, *n* = 19) and P8–P9 (*n* = 34). The anti AP has comparable amplitude in the two populations (77.9 ± 1.71 mV vs. 82.98 ± 2 mV; ns) as well as the resting membrane potential (Em; Table 1). The latency of the anti AP was significantly reduced in older motoneurons (2.40 ± 0.29 ms vs. 1.18 ± 0.13 ms; *p* = 0.0008) due to ongoing motor axon myelination during this postnatal period.

**Table 1:** **Changes of electrical properties of lumbar motoneurons with age in postnatal mice**.

	**P3–P5**	**P8–P9**	
Em (mV)	−68.89 ± 1.02 (19)	−70.03 ± 1.23 (34)	ns
Rin (MΩ)	19.36 ± 1.42 (19)	16.2 ± 0.82 (34)	*
Anti AP latency (ms)	2.40 ± 0.29 (13)	1.18 ± 0.13 (24)	***
Anti AP amplitude (mV)	77.9 ± 1.71 (13)	82.98 ± 2 (24)	ns
Anti AP time to Peak (ms)	1.39 ± 0.13	1.25 ± 0.06 (24)	ns
Anti AP half-width (ms)	1.37 ± 0.08	1.08 ± 0.04 (24)	**
Rheobase (nA)	0.87 ± 0.11 (19)	1.76 ± 0.2 (31)	**
AP threshold (mV)	−50.2 ± 1.73 (9)	−47.88 ± 1.24 (26)	ns
AP amplitude (mV)	62.08 ± 1.68 (12)	68.11 ± 1.76 (11)	*
Time to peak (ms)	1.19 ± 0.07 (12)	0.99 ± 0.05 (11)	ns
Half-width (ms)	1.28 ± 0.11 (12)	0.97 ± 0.03 (11)	**
AP Max depol	123 ± 8.69 (12)	149 ± 7.38 (11)	*
slope (mV/ms)			
AP Max repol	−54.81 ± 5.34 (12)	−72.12 ± 2.90 (11)	**
slope (mV/ms)			
Gain (Hz/nA)	23.80 ± 2.05 (13)	25.63 ± 1.60 (11)	ns
AHP duration (ms)	93.27 ± 7.40 (8)	96.63 ± 6.61 (11)	ns
AHP amplitude (mV)	5.01 ± 0.72 (8)	5.26 ± 0.50 (11)	ns
AHP time max	13.90 ± 1.94 (8)	10.00 ± 1.37 (11)	ns
amplitude (ms)			
AHP 1/2 duration (ms)	47.10 ± 4.33 (8)	36.78 ± 3.01 (11)	*
AHP decay time (ms)	79.61 ± 8.27 (8)	82.74 ± 5.40 (11)	ns
AHP 1/2 decay time (ms)	34.16 ± 3.50 (8)	27.78 ± 2.01 (11)	*

P3–P5, postnatal days 3–5; P8–P9, postnatal days 8 and 9; Em, resting membrane potential; Rin, Input resistance, anti AP, antidromic action potential, latency, latency of AP elicited by electrical stimulation of the L5 ventral root; Time to peak, time needed for potential to rise from spike threshold to maximum value. Half width, time spent by the potential >50% of the maximum amplitude of action potential; Rheobase, lowest intensity of current injected through the electrode to elicit an action potential; AP threshold, voltage threshold measured at the foot of the action potential evoked by intracellular current injection; AP amplitude, amplitude of action potential measured between the foot and the peak; time to peak, measured as the time needed for the potential to reach the peak; Max depol slope, maximum slope of the depolarizing phase of action potential; Max repol slope, maximum slope of the repolarizing phase of action potential; Gain, slope of the steady state frequency-intensity curves measured during injection of long-duration pulse currents (800 ms to 1 s. AHP, Afterhyperpolarization, half duration of AHP is significantly shortened with age as well as half decay time. All values are means ± sem. The number of motoneurons is indicated between brackets after each mean value and sem. ns, non-significant difference; *p < 0.05, **p < 0.01; ***p < 0.005. Nonparametric permutation exact test.

As expected, the mean Rin was lower in the oldest motoneurons (16.2 ± 0.82 MΩ, *n* = 34, P8–P9) compared to the mean Rin in the P3–P5 population (19.36 ± 1.42 MΩ; *n* = 19, *p* = 0.023). However, the Rin stabilizes between P8 and P9 (Figure 2A). At the same time, the rheobase (minimum current to elicit an AP) increases two times in motoneurons from 0.87 ± 0.11 nA (*n* = 19) to 1.76 ± 0.2 nA (*n*= 31) between P3 and P8 (Table 1; Figure 1B, *p* = 0.009). The mean rheobase is significantly higher at P6/P7 (1.53 ± 0.19 nA, *n* = 20; *p* = 0.0036) compared to that in P3–P5. Indeed, a significant and negative correlation exists between the rheobase and the Rin (Figure 2C; *r* = 0.16, *p* = 0.023, Pearson’s correlation test) but no significant correlation was found between the gain and the Rin (Figure 2D; *R*^2^ = 0.09, *p* > 0.05). The mean rheobase was significantly lower at P9 (1.19 ± 0.2, *n* = 12) compared to that at P8 (2.12 ± 0.25, *n* = 19). Thus the progression of the rheobase stopped between P8 and P9 in lumbar motoneurons.

In twelve motoneurons intracellularly stained and fully reconstructed, significant correlations were found between the Rin and the total dendritic surface area (Figure 2E, *r* = −0.67; *p* = 0.016; Pearson’s correlation test) and between the rheobase and the total dendritic surface area (Figure 2F, *r* = 0.63; *p* = 0.028; Pearson’s correlation test) confirming that the largest motoneurons in the lumbar cord have the lowest Rin and the highest rheobase also during postnatal development. However, we noticed that some motoneurons with similar total dendritic surface area (40,000 μm^2^) may have different Rin ranging from 10 to almost 30 MΩ (Figure 2E). Others motoneurons with an Rin around 10–15 MΩ also display different dendritic surface areas (30,000–60,000 μm^2^).

### Changes in AP Shape

As summarized on Table 1, significant differences on the AP shape were found between the two groups of ages. Peak amplitude of the AP was increased from 62.08 ± 1.68 mV to 68.11 ± 1.76 mV (*p* = 0.028) together with the maximum depolarizing speed which was accelerated from 123 ± 8.69 mV.ms^−1^ to 149 ± 7.38 mV.ms^−1^ with age (*p* < 0.01). Thus younger motoneurons have spikes with lower amplitude and slower time course. No changes with age were seen in AHP amplitude and total AHP duration between P3 and P9. However, AHP half-duration was shorter and half decay time faster in the P8–P9 group (Table 1). Others parameters did not change during this short period of time such as spike threshold and the gain of motoneurons measured in the steady state of the discharge frequency.

### Discharge Properties

**Figure 3:**
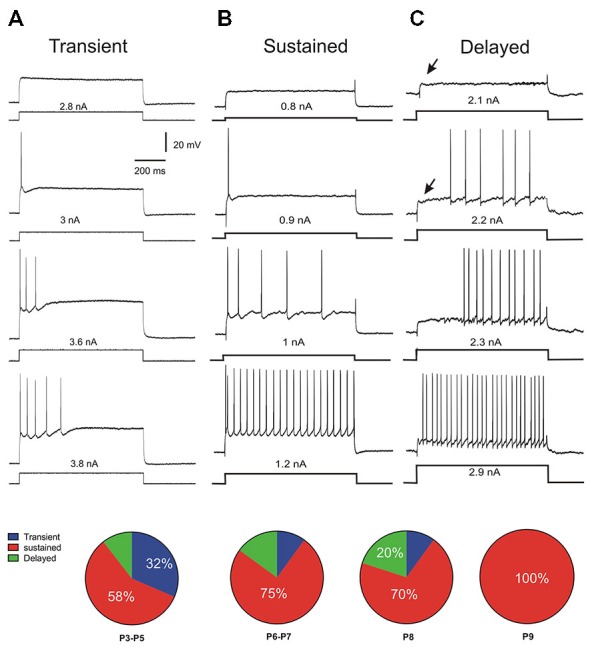
**Discharge firing patterns and distributions with age**. Three different types of discharge firing patterns were found in lumbar motoneurons (*n* = 70) at different postnatal ages in response to rectangular current injection. The transient discharge **(A)** is characterized by a short burst of spike. The sustained pattern **(B)** starts by an early spiking followed by a burst and discharge firing maintained during the whole pulse. The third pattern is called delayed onset discharge firing **(C)** the arrow indicating a late depolarization. At P3–P5, one third of motoneurons still present a transient discharge and the motoneuron is not able to fire APs up to the end of the pulse as illustrated in **(A)** (see pie chart). In older animals the number of motoneurons presenting this pattern decreases up to P9. At that age, there is no more transient firing but all motoneurons exhibit a sustained discharge firing pattern **(B)**. Between P3 and P8 a fraction of motoneurons has a delayed onset firing pattern which disappears at P9.

During the development of spinal motoneurons, different patterns of discharge have been previously described (Vinay et al., 2000a; Mentis et al., 2007; Pambo-Pambo et al., 2009; Leroy et al., 2014). Only recently a delayed onset firing pattern was detected in lumbar motoneurons in slice preparation (Pambo-Pambo et al., 2009; Leroy et al., 2014). We then used intracellular injection of depolarizing constant current pulses (pulse protocols, see methods) to analyze in details the discharge firing pattern of the motoneurons in the whole brainstem-spinal cord preparation. Three different firing patterns were clearly identified during this period of maturation (P3–P9) according to the mode of discharge firing which was transient (Figure 3A), sustained (Figure 3B) or delayed (Figure 3C) in the different motoneurons. Figure 3A illustrates the typical pattern of discharge of transient firing cells. The motoneuron fired a single spike or a burst of spikes and the discharge firing did not last during the entire pulse but a few hundreds of ms, only. This transient firing was recorded in 32% of motoneurons at P3–P5 (pie chart in Figure 3). This ratio is in agreement with previous report on extensor motoneurons in the neonate rat (Vinay et al., 2000a). The transient firing pattern was not observed in motoneurons from animals older than P8 (see pie chart). The second group called sustained firing also displayed an early AP at rheobase and then several APs appeared with increasing current intensities (Figure 3B). The instantaneous discharge frequency progressively increased and the motoneuron was able to fire continuously up to the end of the pulse (Figure 3B; 1.2 nA). A third group is composed of motoneurons that display a delayed onset firing. The delayed onset firing (Figure 3C) corresponds to the late bursting motoneurons previously described in spinal motoneurons (Pambo-Pambo et al., 2009). As illustrated on Figure 3C, they were characterized by a delayed trigger of the AP. At potentials below spike threshold, an initial transient small overshoot in voltage response to the current pulse was observed (arrow on Figure 3C, upper traces). It was followed by a slow rising, late depolarization. This delayed onset firing was seen in 20% of motoneurons in our sample at P8 but was not detected at P9 (*n* = 12). The distribution of the different firing patterns is illustrated in pie charts in Figure 3. Most motoneurons exhibit an early and sustained discharge (as in 3B) at all postnatal ages.

**Figure 4:**
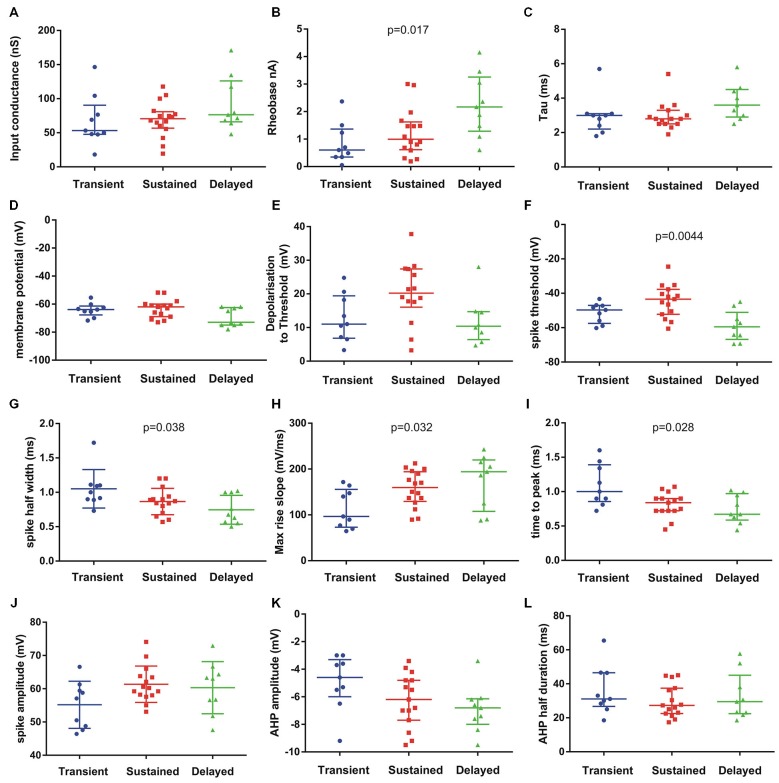
**Passive and active electrical properties of postnatal mouse lumbar motoneurons (*n* = 33) in three subgroups defined by their discharge firing pattern**. Fourteen electrical properties were compared (12 illustrated). Among them, several electrical properties show significant differences in the medians as indicated on each graph (Kruskal–Wallis test). The delayed subgroup has the highest conductance **(A)** rheobase **(B)** and time constant **(C)** suggesting they represent the largest motoneurons. The resting membrane potential (Em) **(D)** was hyperpolarized in this subgroup (delayed) but the spike voltage threshold was the lowest **(F)**. The spike voltage threshold and the depolarization to threshold were the highest in the sustained subgroup **(E,F)**. The AP was the shortest in the delayed population (half width in **G**) and the fastest **(H,I)** compared to the two others groups. The AP amplitude was similar in sustained and delayed subgroups but lower in the transient group **(J)**. The AHP amplitude was smaller in the transient subgroup and larger in the delayed subgroup **(K)** whereas the medians of AHP half durations were comparable in the three subgroups **(L)**. Bars indicate median and quartile. Statistical significance for three populations: Kruskal–Wallis exact test.

We then analysed the electrical properties of the subgroups. We focused on the three patterns of discharge firing in motoneurons from P6–P9 animals. We analyzed 14 passive and active electrical properties (Figure 4). The motoneurons with delayed onset firing pattern have the highest rheobase, input conductance and time constant (Figures 4A–C) suggesting that they are the largest motoneurons. Others parameters show significant differences between the three groups such as the spike threshold (*p* = 0.0044, Kruskall–Wallis test), spike half width (*p* = 0.038, Kruskall–Wallis test), max rise slope (*p* = 0.032, Kruskall–Wallis test) and time to peak (*p* = 0.028, Kruskall–Wallis test) of the AP. The maximum decay slope also shows statistically significant difference between the three groups (not illustrated). All spike parameters (time to peak, max rise slope) indicate that the delayed firing type do have the fastest AP. The AHP parameters did not show significant differences although there is a tendency in the AHP amplitude to be larger in the delayed group (Figures 4K,L). The gain of the motoneurons measured at the steady state firing frequency (in the last 500 ms of the current pulse) was also higher in the delayed onset firing group compared with the gain in the sustained firing group (delayed : 37.2 ± 2.5 Hz/nA, *n* = 9; sustained : 27.7 ± 3.4 Hz/nA, *n* = 15; *p* = 0.024; non parametric permutation exact test).

### Firing Behavior on Slow Triangular Current Ramps

**Figure 5:**
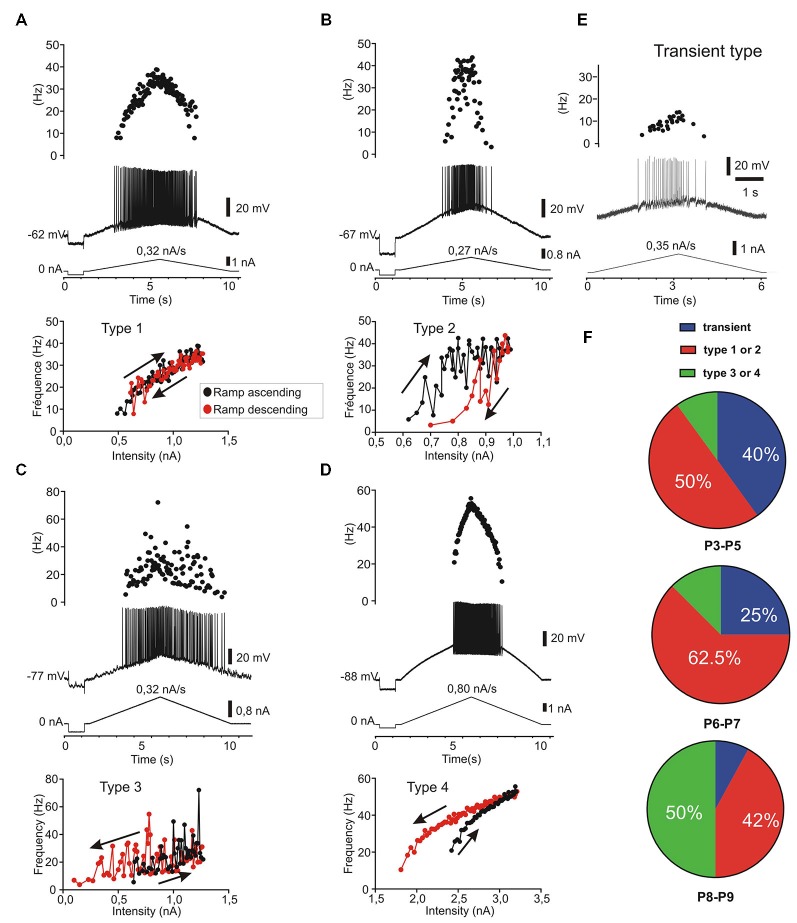
**Five patterns of discharge recorded in response to current ramp stimulation in postnatal lumbar motoneurons (*n* = 38)**. The four classical types **(A–D)** as described in adult motoneurons (Bennett et al., 2001) are present and a fifth type called transient, since no discharge firing, or only a few spikes (<5), could be evoked during the descending phase **(E)**. **(A)** type 1: linear F-I relationship where the firing frequency curves overlapped on the ascending and descending phases. **(B)** type 2: clockwise hysteresis pattern where the instantaneous frequency is lower in the descending phase for the same current intensity. **(C)** type 3: Linear F-I relationship with sustained firing in the descending phase. **(D)** type 4: Counter clockwise hysteresis where the frequency is higher during the descending phase; **(E)** type 5: transient discharge during the ascending phase with usually no discharge or only a few spikes in the descending phase. In this case which is frequent before P5 (40%), it was not possible to plot an F-I curve during the descending ramp. **(F)** distribution of the five discharge patterns according to the postnatal ages. Note that types 1 and 2 are the predominant types before P8 (*n* = 15/26) whereas the types 3 and 4 are most frequent at P8–P9 (*n* = 6/12) but rare before P8 (*n* = 3/26).

Firing pattern was further characterized using increasing and decreasing (triangular) slow current ramps as described previously (Hounsgaard et al., 1988; Amendola et al., 2007; Pambo-Pambo et al., 2009). The F-I plots revealed differences in discharge patterns which could be classified in five types according to the frequency response on up and down going ramps. The four classical types already described in motoneurons from adult (Bennett et al., 2001) or neonate (Amendola et al., 2007) rodents are illustrated as followed: linear (Figure 5A), clockwise hysteresis (Figure 5B), prolonged sustained (Figure 5C) and counterclockwise hysteresis (Figure 5D). A fifth type was defined by the lack or the quasi-absence of discharge firing during the descending phase of the ramp at early postnatal ages (Figure 5E). This fifth type was present in 40% of motoneurons at P3–P5, in 25% of motoneurons at P6–P7 and in less than 10% at P8–P9 (Figure 5F). At an early postnatal age, type 1 (linear) and type 2 (clockwise) were predominant whereas the number of motoneurons exhibiting type 3 and type 4 markedly increases at P8–P9. Type 3 and type 4 discharge patterns, displaying a sustained firing during the down going ramp and a counter-clockwise hysteresis, respectively, was observed in 50% of motoneurons in older animals whereas the number of motoneurons exhibiting a non-linear electrical behavior during ramp current injection was <20% before P8. The emergence of types 3 and 4 discharge patterns correspond with the period of maturation of L-type calcium channels in mouse motoneurons (Carlin et al., 2000).

We found no correlation between the different groups of motoneurons defined by step of current and those defined by triangular ramp of currents with the exception of the transient firing patterns. In other words, the delayed onset firing group and the sustained firing group both contain motoneurons of different types 1–4 in the Bennett’s classification following ascending descending ramps of current (Bennett et al., 2001).

## Discussion

In this work, we show rapid changes in electrical properties of postnatal mouse lumbar motoneurons. We defined three different groups of lumbar motoneurons according to their discharge firing patterns and differences in several electrical properties. The delayed onset firing type has the characteristics of the largest motoneurons whereas the transient type is the less mature group of motoneurons and contains mainly small motoneurons with low rheobase and slow APs. We found that 32% of motoneurons still discharged transiently at an early age (P3–P5) whereas some motoneurons exhibit a delayed onset firing pattern up to the second postnatal week. A majority of motoneurons have a sustained firing at all ages between P3 and P9. The sustained firing is present in all motoneurons at P9. The results also show that a counter clockwise hysteresis and/or a prolonged sustained firing in response to current ramp (types 3 and 4) emerge at P8–P9 corresponding to the maturation of L type calcium channels in dendrites of mouse motoneurons. Dendrites of postnatal motoneurons mainly elongated during this short period of time. Although most morphological parameters were not significantly different between P3 and P9 (Filipchuk and Durand, 2012), a significant correlation exists between the size of the dendritic arborizations and the Rin or the rheobase of motoneurons (Figures 2E,F).

### Postnatal Changes in Electrical Properties of Lumbar Motoneurons

The electrical properties of spinal motoneurons have been well documented in the neonate rat (Seebach and Mendell, 1996; Vinay et al., 2000a, 2002) but only two studies concern neonate mouse spinal motoneurons and the evolution of several passive and active properties (Nakanishi and Whelan, 2010; Quinlan et al., 2011). We show that rheobase, Rin, spike half-width and spike depolarization speed changed significantly in mouse spinal motoneurons between postnatal days 3 and 9. The AP and half decay AHP are found to shorten significantly in duration (Table 1). The shape of AP is modified with a higher speed of depolarization in older animals probably linked with the density of sodium channels (García et al., 1998; Carlin et al., 2008) and a faster repolarization indicating potassium channels maturation (McLarnon, 1995; Gao and Ziskind-Conhaim, 1998; Nakanishi and Whelan, 2010). During the same time, some parameters of electrical properties remain constant such as the membrane potential, AP threshold and amplitude, gain at steady state and after hyperpolarization duration and amplitude (Table 1).

Our results are comparable to those obtained previously in rodents (Fulton and Walton, 1986; Seebach and Mendell, 1996; Vinay et al., 2000a,b; Mentis et al., 2007; Nakanishi and Whelan, 2010; Quinlan et al., 2011).

A few exceptions concerns some parameters such as membrane potentials and Rin (Nakanishi and Whelan, 2010; Quinlan et al., 2011). In the study by Quinlan et al. (2011) membrane potentials were significantly different probably because the younger population of motoneurons started from P0 where motoneurons have more depolarized potentials whereas in our study the younger group was aged between P3 and P5. Surprisingly the mean Rin were not different between the younger and older groups of motoneurons in the study by Nakanishi and Whelan (2010). The slicing procedure and the visual selection of neurons cannot fully explain this result since those by Quinlan et al. (2011) have been also obtained in slice. All other developmental changes in electrical parameters have been described in many other species (Kellerth et al., 1971; Hammarberg and Kellerth, 1975; Navarrette and Vrbová, 1993; Perrier and Hounsgaard, 2000; Vinay et al., 2000b) including human spinal motoneurons derived from embryonic stem cells (Takazawa et al., 2012).

The rheobase current significantly increased during this short time period between P3 and P8 and stabilized between P8 and P9 precisely when the Rin stops decreasing. As already described in neonate rats (Seebach and Mendell, 1996), a significant correlation exists between Rin and rheobase in our sample of developing mouse motoneurons (see Figure 2). We also found a positive correlation between the size of the dendritic arborizations and the rheobase of developing motoneurons (Figure 2). The size of motoneurons (both soma and dendritic arborizations) increases with age although the dendritic arborizations did not increase in complexity during this postnatal period in mice (Li et al., 2005; Filipchuk and Durand, 2012). In fact this is a time for rapid changes in active electrical properties whereas the morphology of motoneurons with their dendritic arborizations growth in a slow and progressive manner.

### Different Patterns of Discharge Firing

In this study, we found 20% of motoneurons with a delayed onset firing pattern. In another set of motoneurons, a maximal proportion of 27% of such delayed firing was reached at an age between P6 and P8 (not shown). This proportion is rather low compared to that (65%) observed in lumbar motoneurons recorded in slice (Pambo-Pambo et al., 2009; Leroy et al., 2014). The delayed onset firing pattern is due to transient outward potassium currents as shown by blockade with apamin and TEA (Takahashi, 1990; Russier et al., 2003; Pambo-Pambo et al., 2009). Thus the major difference between both ratios obtained in the different spinal cord preparations might be linked to modulation by supraspinal descending pathways controlling potassium conductances and the absence of such control in slice preparations (McLarnon, 1995; Perrier and Hounsgaard, 2000). The number of motoneurons with such firing pattern might not have been under evaluated due to short current pulse (which are used in most studies) as suggested in recent work (Leroy et al., 2014) since comparable current pulses (1 s) were applied in our study in slice preparation (Pambo-Pambo et al., 2009) and in the present study. The delayed onset firing was not seen in spinal motoneurons from neonate rat probably due to the level of the brainstem section and/or the age of the animals. We found a transient expression of the delayed onset firing type between P3 and P8 (Figure 3) which is rather close to results obtained in rat abducens motoneurons (Russier et al., 2003) and neonate oculomotor (Nieto-Gonzalez et al., 2007). Although we did not investigate younger animals than P3, it is noteworthy that the delayed onset firing type was never seen in large studies performed at P0–P2 in neonate rat (Vinay et al., 2000a,b, 2002). In most work performed on older animals (>P8), the delayed onset firing pattern was not described in spinal motoneurons (Miles et al., 2005; Delestrée et al., 2014) but see Zhu et al. (2012). The delayed onset firing pattern is also present in adult rat facial motoneurons (Nishimura et al., 1989) but not in adult abducens and oculomotor motoneurons (Durand, 1989a,b).

The differences of several parameters in the electrical properties of the three groups of motoneurons likely reflect differences in maturation as suggested by APs parameters (Figure 4). Indeed our results show that the motoneurons with transient firing pattern are populations of immature cells whose properties will change after P8. The transient firing pattern was present in most brainstem and spinal motoneurons in neonate animals (Vinay et al., 2000b; Nieto-Gonzalez et al., 2007) but see Leroy et al. (2014). The transient firing type was seen in more than half of extensor motoneurons at P0–P2 and in 30% at P3–P5, whereas the sustained firing type represents 70% in extensor and 100% in flexor motoneurons at P3–P5 (Vinay et al., 2000a). The reason why the transient firing was not found in some studies might be due to recording of a majority of flexor motoneurons.

We can notice that a transient firing type is still present in adult brainstem motoneurons (Durand, 1989a; Nishimura et al., 1989; Nieto-Gonzalez et al., 2007) and in adult zebrafish (Ampatzis et al., 2013) but not in mammalian spinal motoneurons (Delestrée et al., 2014).

It is difficult to speculate on the presence of these discharge patterns in the different types of motoneurons and species. Our study shows that both delayed and transient firing patterns disappear at P9 in mouse lumbar motoneurons. At that age there is still gap junctions between motoneurons as shown by multiple staining after a single intracellular injection (Amendola and Durand, 2008). Gap junctions between motoneurons and polyinnervation of muscular fibers disappear between the second and the third postnatal week (Navarrette and Vrbová, 1993; Kopp et al., 2000; Mentis et al., 2002; Vinay et al., 2002). The complementarity of the delayed and transient discharges might insure an asynchronous firing in the same pool promoting synapse elimination (Buffelli et al., 2002, 2004). The transient and the delayed onset types might represent the last motoneurons innervating common muscle fibers. We speculate that the large motoneurons with delayed onset type will prefer fast twitch fibers whereas the motoneurons with transient type will win the innervation of slow muscle fibers. If motoneurons are recruited together, it may also represent a protection for the muscular fibers against strong activation by two motoneurons. On the other hand, the different discharge pattern might contribute to enhance the phenotypic differences among fast and slow muscle fiber types by differentially regulating transcription in a use dependent manner (Rana et al., 2009).

### Persistent Inward Current and Ramp in Motoneurons

Using ramp of current we found an increase with age in the number of motoneurons with non-linear behaviors (types 3 and 4). They represent up to 50% of motoneurons at P8–P9 but only 10% at P3–P5 (Figure 5). We found no correlation between the different types of motoneurons based on their firing properties defined by pulse steps or triangular current stimulations except for transient types. Mouse spinal motoneurons are endowed with functionally mature calcium channels in the second postnatal week (Carlin et al., 2000).

The non-linear behavior may originate from sodium and/or calcium persistent inward currents (Schwindt and Crill, 1980; Perrier and Hounsgaard, 2000; Heckman et al., 2008). Recently, it was found that bistable behaviors are unmasked 1 week after birth in 80% of motoneurons when the temperature was raised >30°C (Bouhadfane et al., 2013). However all our experiments have been performed at a lower temperature (24–25°C). Our results are compatible with those describing the development of L type calcium channels in the mouse (Jiang et al., 1999b). This also parallels the maturation of functional behaviors in rodents (Vinay et al., 2005), mice begin to weight-bear and walk at P9–P10 (Fox, 1965; Jiang et al., 1999a; Amendola et al., 2004).

### Importance of Our Findings for Future Studies on ALS

Our results show that the rheobase, input conductance and gain of motoneurons are the highest in the delayed firing group. These results are in agreement with those suggesting that motoneurons with delayed onset firing pattern correspond to the fast motoneurons (Leroy et al., 2014). Indeed it is tempting to speculate that these motoneurons will be part of the future population of fast motoneurons in older animals since a proportion of 30% motoneurons expressing Dlk1, a biophysical marker for fast motoneurons, was recently found (Muller et al., 2014). In addition the delayed onset firing type motoneurons present the more hyperpolarized membrane potential (Figure 4D). Recently, Hadzipasic et al. (2014) identified four types of spinal motoneurons in the adult mice and showed that the fastest firing motoneuron type was lost in a SOD1^G85R^ transgenic mouse model of ALS at 3–4 months of age. Furthermore they found that this population of motoneurons that disappears in SOD1 adult mice was greatly hyperpolarized, which would favour hypoexcitability. We previously showed that lumbar motoneurons from SOD1^G85R^ mice were hypoexcitable very early during the postnatal period having a higher rheobase and lower gain (Bories et al., 2007). It would be important to determine whether the population of motoneurons which degenerate first in adult SOD1 mice corresponds to the hypoexcitable cells detected in the postnatal period. Therefore, it remains to be determined whether the delayed onset firing type is affected in SOD1 postnatal mice. It seems not to be the case in the study by Leroy et al. (2014). However this latter study used the high expressor strain of SOD1^G93A^ mice in which an accelerated maturation of lumbar motoneurons might lead to a different time course in the ALS pathology (Quinlan et al., 2011, 2015). Further longitudinal studies from low expressor strains of SOD1^G85R^ or SOD1^G93A^ mice are needed to elucidate this question.

## Conclusions

We found rapid changes in the progression of electrical properties of mouse lumbar motoneurons between P3 and P9 whereas the morphology of dendritic arborization evolves slowly. A change of rheobase and Rin progressions occurs, with the disappearance of transient and delayed onset firing types following direct current pulse stimulation, and the emergence of types 3 and 4 discharge patterns following direct ramp current stimulation. We conclude that a switch might exist in the electrical properties of mouse lumbar motoneurons around P8–P9 during the maturation of motor behaviors.

## Conflict of Interest Statement

The authors declare that the research was conducted in the absence of any commercial or financial relationships that could be construed as a potential conflict of interest.
